# ChemDoodle Web Components: HTML5 toolkit for chemical graphics, interfaces, and informatics

**DOI:** 10.1186/s13321-015-0085-3

**Published:** 2015-07-16

**Authors:** Melanie C Burger

**Affiliations:** Biomedical Communications, Department of Biology, University of Toronto Mississauga, HSC 308, 3359 Mississauga Road, Mississauga, ON L5L 1C6 Canada; Imagineeringart.com Inc., 208 Bloor Street West Suite 300, Toronto, ON M5S 3B4 Canada

**Keywords:** ChemDoodle Web Components, Chemical graphics, Animations, Cheminformatics, HTML5, Canvas, JavaScript, WebGL, Structure editor, Structure query

## Abstract

ChemDoodle Web Components (abbreviated CWC, iChemLabs, LLC) is a light-weight (~340 KB) JavaScript/HTML5 toolkit for chemical graphics, structure editing, interfaces, and informatics based on the proprietary ChemDoodle desktop software. The library uses <canvas> and WebGL technologies and other HTML5 features to provide solutions for creating chemistry-related applications for the web on desktop and mobile platforms. CWC can serve a broad range of scientific disciplines including crystallography, materials science, organic and inorganic chemistry, biochemistry and chemical biology. CWC is freely available for in-house use and is open source (GPL v3) for all other uses.

**Graphical abstract:**
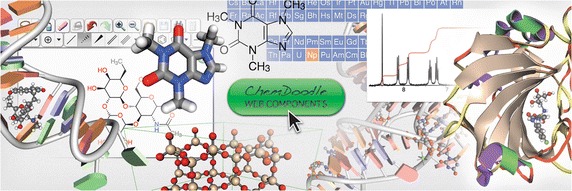
Add interactive 2D and 3D chemical sketchers, graphics, and spectra to websites and apps with ChemDoodle Web Components.

Add interactive 2D and 3D chemical sketchers, graphics, and spectra to websites and apps with ChemDoodle Web Components.

## Introduction

How we communicate chemical information is increasingly technology driven. Learning management systems, virtual classrooms and MOOCs are a few examples where chemistry educators need forward compatible tools for digital natives. Companies that implement emerging web technologies can find efficiencies and benefit from competitive advantages. The first chemical graphics toolkit for the web, MDL Chime, was introduced in 1996 [1]. Based on the molecular visualization program RasMol, Chime was developed as a plugin for Netscape and later for Internet Explorer and Firefox. In 2004 Jmol, a Java applet [2], was released to replace Chime and provide an open source and operating system independent solution to the growing number of web browsers. JME, a molecular editor [3], was later integrated into Jmol to add chemical structure upload and editing functionality. In 2007 however, the hardware landscape changed dramatically with the introduction of mobile devices that did not support third party plugins such as Flash or Java applets. Mobile browsers did support HTML5, which opened the door to web applications built with only HTML, CSS and JavaScript (JS), such as the ChemDoodle Web Components.

## Review

### The ChemDoodle Web Components technology stack and features

The ChemDoodle Web Components library, released in 2009, is the first chemistry toolkit for structure viewing and editing that is originally built using only web standard technologies, HTML5, CSS, and JS, and is accordingly supported by all modern desktop and mobile browsers. CWC is made available under the free and open source, GNU GPLv3 license and is accompanied by detailed documentation and commercial support packages. The CWC source code follows JS best practices to ensure maintainability and cross-compatibility with other libraries and frameworks.

Using web standard technology has a number of advantages over 3rd party plugins like Java applets and Flash:

- installation or updates are not required since JS is enabled in browsers by default,
- JS applications are quick to load without the overhead of 3rd party plugins,
- there are fewer security concerns since the code is sand-boxed in the browser,
- the deep integration of HTML, CSS and JS in the browser creates a seamless user experience, and
- the ChemDoodle Web Components library can be loaded and displayed wherever a HTML5 engine is available, including WYSIWIG text editors, Apple’s Cocoa development kit, and mobile app webviews.

Beyond rendering 2D and 3D chemical graphics, the library also provides access to cheminformatics algorithms, chemical file input/output and manipulation, and a toolset for chemistry web application development through a component system that gives the library its name. The components of the CWC library are specialized HTML5 <canvas> classes that expose a high-level API for quick loading and viewing of chemical data, as well as providing utility functions and multi-device event handling. When first released, CWC comprised of 6 components: the Viewer, Rotator, Transformer, MolGrabber, File Loader, and Doodler (pre-cursor to Sketcher) components. Averaging a yearly version release cycle, CWC has now grown to 20 components including ones for displaying chemical spectra, 3D WebGL graphics, and animations. The latest update, version 7, introduces new 3D features including Pipe and Plank protein models, full support for high DPI and retina display devices, query interface tools for advanced chemical searches, and structure spectrum correlation utilities.

### Usage

**Figure 1:**
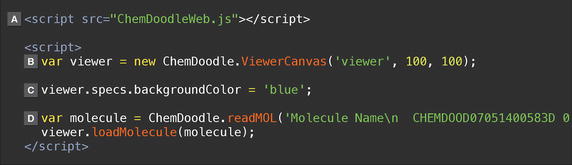
JavaScript code for creating an instance of a Viewer component on a web page. The ChemDoodle Web Components library is *A* included in the web page using the <script> tag. *B* The Viewer component is initialized with the ‘new’ keyword and is provided an identifying name, and dimensions of width and height. *C* The style of the Viewer <canvas> and its contents can be optionally customized from default settings. *D* Molecular data is loaded into the viewer. Here, the contents of a MOL file are first read by CWC, converted into a molecule object, and then loaded in the component. The molecule is automatically centered and scaled to fit the <canvas>.

**Figure 2:**
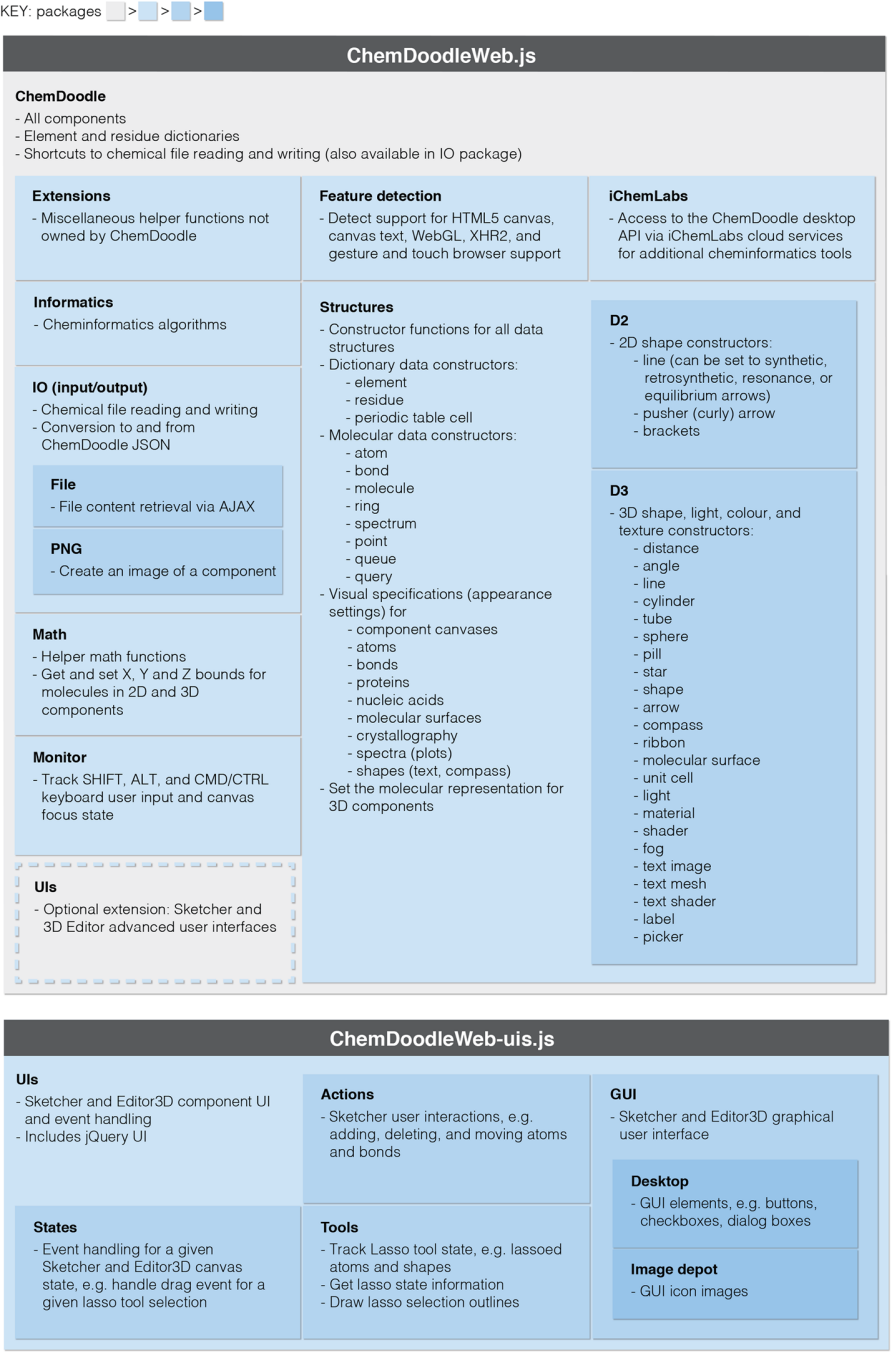
Overview of the ChemDoodle Web Component library API. The ChemDoodle library is organized into hierarchical packages that contain related functions. Loading the optional ChemDoodle-UIs package adds the Sketcher and Editor3D components extensions to the CWC library. Further information about the API can be found the in the CWC documentation.

A ChemDoodle Web Component is implemented in four basic steps (Figure 1). First, the ChemDoodle Web Components library is referenced within the HTML document. Second, the component is instantiated on the page within a JavaScript code block. The appearance of the component can be customized in an optional third step. Finally, molecular data is loaded into the component either by creating a molecule manually, or by fetching ChemDoodle JSON or the contents of a MOL file or other molecular structure file. The components are “chemically intelligent”: chemical data loaded in components can be later handled or altered with scripting. Support for building components is also available in the ChemDoodle v7 desktop application. Component code can be exported through the “Generate ChemDoodle Web Component” dialogue screen, however, advanced users will prefer to use the CWC API (Figure 2) for full control over component appearance and behavior. Tutorials for using the CWC API are provided on the CWC website, web.chemdoodle.com.

### 2D graphics

The 2D graphics engine in the ChemDoodle Web Components is based on HTML5 <canvas> technology. The <canvas> tag creates a bitmap drawing surface in an HTML document that is controllable with JS. CWC abstracts the <canvas> interface and provides a uniform codebase for drawing a wide range of 2D chemical graphics within 2D CWC components:

- When displayed, atom labels can show standard element labels or any alternative symbol.
- Ionic/zero order, half, single, double, triple, and resonance chemical bond types, and protruding and recessed stereochemical bonds are available.
- Charges, radicals and lone electron pairs are positioned automatically on attributed atoms.
- Advanced query notation can be displayed on atoms or bonds to represent sets of molecules.
- Synthetic, retrosynthetic, resonance and equilibrium arrows can have text placed above or below them.
- Single headed (one electron), double headed (two electron) and middle merging pusher (curly) arrows can be added between selected atoms and bonds. Arrows flow around existing structures automatically.
- Customizable brackets can be placed around part of or a whole molecule to indicate global charges, multiplicity or polymer units.

In addition to molecular structure graphics, spectrum components can display NMR, IR, UV/Vis and mass spectrometry spectra. Control over the plot colors, domain, range, gridlines, tick marks, and title and axes label properties is provided, and integration curves can be generated for NMR spectra. An interactive periodic table component is also available.

**Figure 3:**
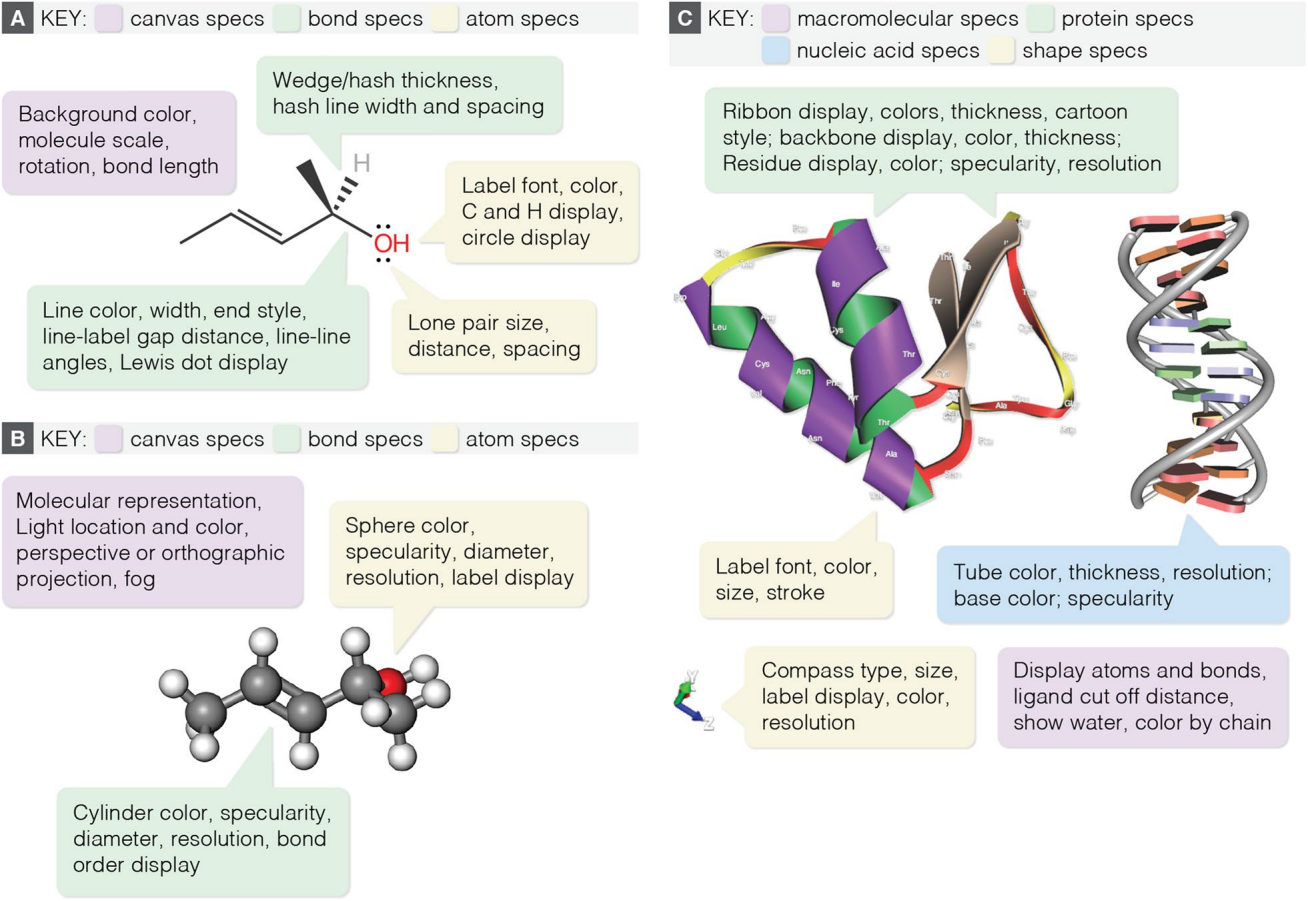
Summary of visual specification settings for small and macromolecules. The CWC visual specifications can be customized to create unique styles for* A* 2D chemical graphics in 2D components *B* 3D chemical graphics in 3D components, and *C* macromolecules such as proteins and nucleic acids and shapes in 3D components.

While the ChemDoodle desktop application offers complete manual control over graphics placement and position, CWC is optimized for quick communication by defining algorithms that help to automatically layout graphics. For example, lone pairs and implicit hydrogens are automatically placed on attributable elements, standard bond angles are used by default, reaction equations are spaced evenly, and component contents are scaled up or down to fit the <canvas> dimensions. Styling of graphical elements can be highly customized from default settings via the CWC visual specifications API however. The visual specifications cover standard settings such as label font and color, and bond length and width, but also offer control over more unique settings such as lone pair size, label buffer (padding) size, dash length and spacing for dashed bonds, and much more (Figure 3A). A large number of molecular representation styles can be achieved by customizing the visual specifications, such as the popular ACS Document 1996 style. Conveniently, the 2D and 3D visual specifications can be defined globally to ensure a consistent, site-wide appearance, assigned to a specific component, or defined locally on individual atoms or bonds.

### 3D graphics

The 3D graphics engine is also based on the HTML5 <canvas> element, but uses the 3D WebGL context instead of the 2D context. WebGL is a graphics card technology that enables hardware accelerated rendering of graphics through the OpenGL pipeline. CWC provides a codebase for producing a large range of 3D graphics with 3D components:

- A text system can display labels for atoms and residues or any arbitrary text.
- Molecular representations include ball-and-stick, stick, van der Waals spheres (CPK), wireframe, and line.
- Ionic/zero order, half, single, double and triple bonds are available.
- Nucleic acids appear as ladder and rung representations.
- Proteins can be displayed as backbone, ribbon, or pipe and plank representations. Ribbon and backbone representations can be colored by chain or by residue using a number of different color presets: amino, shapely, polarity, acidity, and rainbow.
- Water molecules can be displayed as spheres or stars, or hidden.
- Crystallographic structures are shown in periodic unit cells, and can be built into supercells.
- 3D shapes for distance, angle, and torsion measurements, and a compass to indicate current orientation can be added to 3D scenes.

The visual specifications for 3D graphics, like those for 2D graphics, provide broad control over the appearance of the scene, including atom, bond and residue size and material; light position and color; and fog effects (Figure 3B, C). In addition, the scene can be displayed by a perspective or orthographic projection. Display of 2D and 3D graphics on high-resolution displays, such as the Apple Retina display, is fully supported by CWC.

### Interfaces

While the ChemDoodle Web Components offers high quality graphics output, it is mainly a toolkit for producing advanced interfaces for chemical data. Each ChemDoodle Web Component is configured to handle chemical data in a particular way, requiring graphics display, user interaction, animation, and/or other technical systems. For example, molecular data loaded into a Viewer component is displayed as a static 2D Lewis structure drawing, whereas the same data in a Rotator3D component appears as a 3D rotating molecule. The main use cases for the CWCs are:

- rendering different types of chemical information, e.g. chemical structures and spectra,
- providing a specific user experience, e.g. rotating and scaling a molecule, visualizing a process with an animation, or highlighting peaks from spectra,
- enabling advanced user input, e.g. sketching molecules, and
- rendering 3D scenes using WebGL, e.g. to visualize a complex protein structure in 3D.

Because CWC is open source, it is possible to modify the source code to create custom component functionality or create novel component plugins. The component framework is designed to be composable to facilitate rapid development. There are four base classes that form the building blocks of the twenty components: the Canvas, Canvas3D, AnimatorCanvas, and SpectrumCanvas classes. These base classes provide core functions such as drawing molecules to <canvas> instances and event handling for desktop and mobile devices. Each component takes one or more of the four base classes as its prototype and then adds additional properties to create custom functionality. For example, the Rotator component is implemented by extending the Canvas and AnimatorCanvas classes. In the same way, developers can extend core building blocks to get basic behavior ‘for free’ and focus on creating desired component functionality instead.

### Advanced interfaces

CWC provides two advanced interfaces as additional plugins: the Sketcher component, a fully functional 2D chemical sketcher, and the Editor3D component, an interface for exploring 3D molecular models. Of the CWC components, the Sketcher component is the most widely used, having the largest number of applications. Structures drawn in the sketcher can be saved as images, exported to another component, used to query chemical databases, or perform calculations. Three variations of the Sketcher component exist: a single molecule sketcher with a simplified interface, a full-featured sketcher for drawing complex figures, and a query sketcher for chemical structure searches. The Editor3D component shares a number of features with the Sketcher, including ‘Open’, ‘Save’, ‘Search’ and ‘Calculate’ functionality, allowing users to load structures and obtain basic chemical property data. In addition, the Editor3D component enables distance, angle, and torsion measurements, and creates 3D visualizations for these measurements, which can useful for students learning molecular geometry.

The sketchers and 3D editor are installed separately from other components as a plugin to the CWC library (Figure 2), which avoids extraneous downloads for applications that do not need those functions. As with the other components, the Sketcher and Editor3D interfaces can be used on both desktop and mobile devices.

### Cheminformatics

The ChemDoodle Web Components API includes a wide range of cheminformatics algorithms for handling chemical data. Included are algorithms for deducing bonds and hydrogens, splitting disconnected molecule data structures, copying molecules, and an extensible system for performing calculations and ring searches. These algorithms are based on a common molecule data structure that stores atom, bond and ring data structures in arrays. Molecules can be created manually using these data structures, or built from a data source, such as a MOL file.

CWC has broad file type support for input and output of chemical data: XYZ, PDB, CIF and JCAMP files can be read, and MDL MOLfiles, RXN files and chemical markup language (CML) can be read and written. CWC also ships with its own chemical data interchange format, ChemDoodle JSON, which is a native, light-weight way of storing chemical data. ChemDoodle JSON is the recommended format for working with CWC as it is based on JavaScript and fully supports CWC library features. ChemDoodle JSON can also be exported and read by the ChemDoodle desktop application.

If more functionality is desired, the ChemDoodle Web Components can also access the full desktop ChemDoodle API via iChemLabs cloud services. Through these cloud services, developers can access tools for comparing molecules, calculating properties, accessing databases, working with other formats such as ICL, ChemDraw CDX and SMILES, generating and parsing IUPAC names, simulating spectra and more. Access to iChemLabs cloud services is provided for free to academic customers.

### Lab_3_D.io: a case study

**Figure 4:**
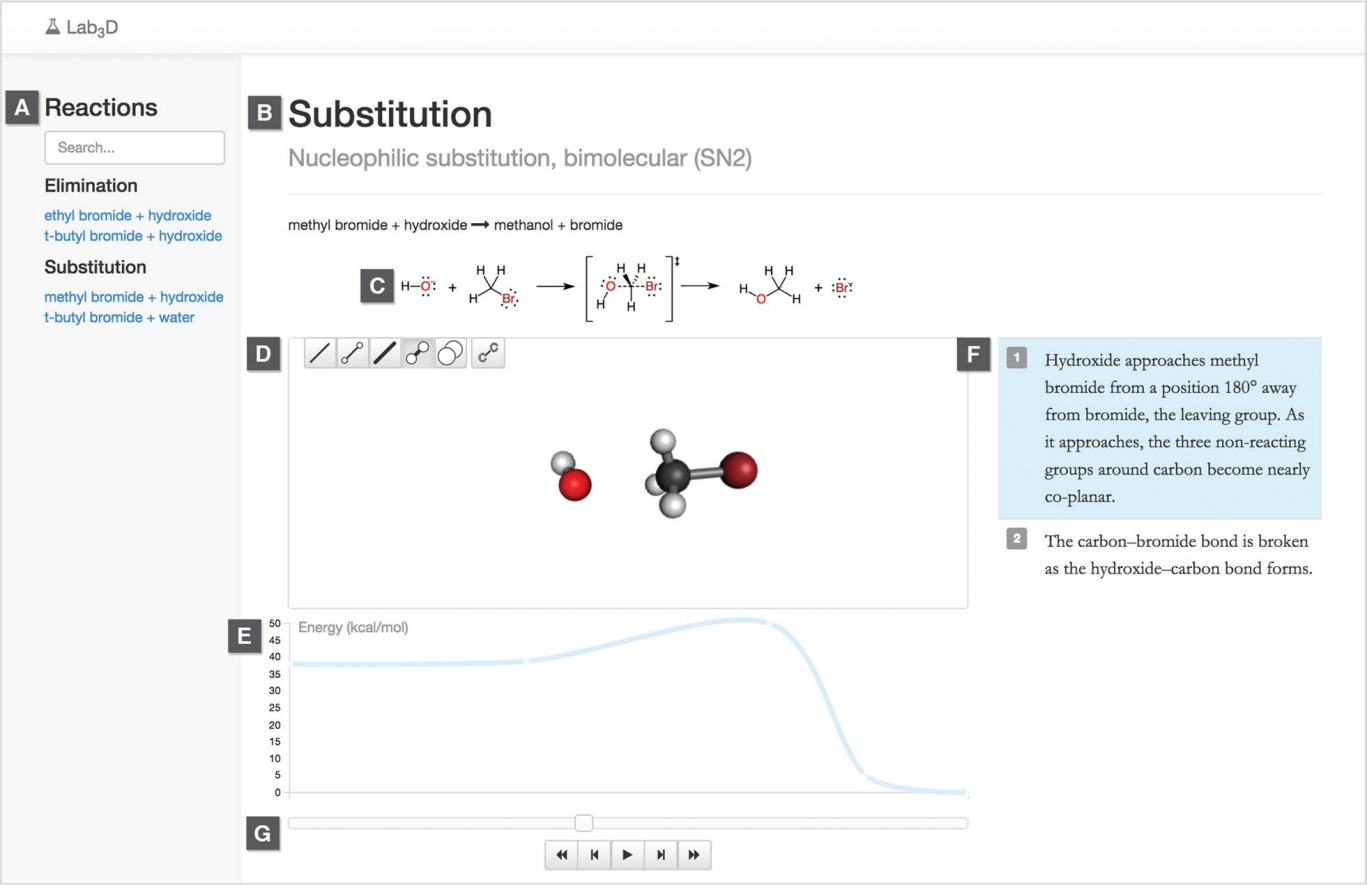
The Lab_3_D user interface. Available reactions are listed in the sidebar (*A*). For each reaction there is *B* a reaction category and subcategory, *C* word and structure equations, *D* a 3D interactive animation of the reaction mechanism, *E* an energy vs. reaction coordinate plot, and *F* contextual descriptions. The animation is controlled via a slider and media controls (*G*).

Lab_3_D.io is an example of how the CWC interfaces can be used to create a chemistry web application. The goal of Lab_3_D.io (Figure 4) is to visualize organic chemistry reactions taught at the college level. The web app dynamically links different types of information that describe a chemical reaction. For each reaction there is

- a word equation,
- a structure equation,
- a plot of energy vs. reaction coordinate,
- contextual descriptions, and
- a 3D interactive animation.

A Viewer component is used to display the chemical structure equation. The equation, drawn in a Sketcher component, was exported as ChemDoodle JSON for loading into the Viewer. Additional graphical elements, such as plus signs and transition state symbols are added by implementing the drawChildExtras function of the CWC API. The Movie3D component is used to show the animation and to expose hooks for additional interactive functionality. Data for the 3D animations were modeled in Spartan ‘10 (Wavefunction Inc.) and GAMESS [4, 5] molecular modeling programs. The MDL SD file containing the sequence of structures is loaded into the Movie3D component. Each molecule loaded into a Movie3D component represents a frame in the animation. To lower molecular modeling time but still produce a smooth animation a script was written that extends the molecular frame array by calculating an average structure for every pair of molecules, and inserting that molecule into the sequence. As the 3D animation plays, the user can explore the mechanism by rotating, translating, and scaling the scene, functionality provided by default by Movie3D. Media controls and a slider leverage the Movie3D component API to offer fine control over the position of the animation. For example, frameNumber, an exposed Movie3D component variable that keeps track of the next animation frame (molecule), is used to update the slider position and highlighted text description as the animation plays. Finally, molecular representation and element labels can be toggled on the fly with scripts that modify the component’s visual specifications settings.

Since CWC is built with web standard technologies, it can be combined with other popular JavaScript libraries for a fully integrated web experience. In the case of Lab_3_D, jQuery (v2.1.1), Bootstrap (v3.1.1) and AngularJS (v1.2.18) are used to create the application framework, jQueryUI (v1.10.4) is used for interface elements, while the plot is created with D3 (v3.4.8). The application framework lays the groundwork for rapid expansion. The website is automatically updated whenever new data are added to a reactions list. At present, only four nucleophilic substitution and elimination reactions are available, but future growth of the website is envisioned through user submission of data. Taken together, Lab_3_D.io shows how the functionality of the ChemDoodle Web Components can be customized and combined with other JS libraries to create, in this example, a novel interactive learning experience.

## Conclusion

ChemDoodle Web Components is a very useful toolkit for chemical graphics display, cheminformatics, and chemistry app development for the web. Because it uses web standard technology, CWC is a good choice for producing new chemistry content for the web. The library’s ease of use, broad browser compatibility, application across numerous chemical disciplines, high-quality graphics, robust and well-documented API, and open source licensing should lead to its popularity in academic, government, and industrial settings.

## Abbreviations

2D: two dimensional
3D: three dimensional
API: application programming interface
CDX: compound index
CIF: crystallographic information file
CML: chemical markup language
CPK: Corey, Pauling and Koltun
CSS: cascading stylesheets
CWC: ChemDoodle Web Components
DPI: dots per inch
GAMESS: general atomic and molecular electronic structure system
GNU: GNU’s not Unix!
GPL: general public license
HTML: hypertext markup language
IR: infrared
IUPAC: International Union of Pure and Applied Chemistry
JCAMP: Joint Committee on Atomic and Molecular Physical Data
JME: Java molecular editor
JS: JavaScript
JSON: JavaScript object notation
KB: kilobytes
LLC: limited liability company
MOOC: massive open online course
NMR: nuclear magnetic resonance
OpenGL: open graphics library
PDB: protein data bank
RXN: MDL molefile reaction format
SMILES: simplified molecular-input line entry system
UV: ultra-violet
WebGL: web graphics library
WYSIWYG: what you see is what you get
